# Prevalence and correlates of cervical squamous intraepithelial lesions among HIV-infected and uninfected women in Central Kenya

**DOI:** 10.11604/pamj.2021.39.44.27182

**Published:** 2021-05-18

**Authors:** Stella Kawira Njagi, Kenneth Ngure, Lawrence Mwaniki, Michael Kiptoo, Nelly Rwamba Mugo

**Affiliations:** 1 Department of Community Health, Jomo Kenyatta University of Agriculture and Technology, Juja, Kenya,; 2 Partners in Health, Research and Development, Thika Section 9, Nairobi, Kenya,; 3 Institute for Tropical Medicine and HIV and AIDS Laboratory, Kenya Medical Research Institute, Nairobi, Kenya,; 4 Sexual Reproduction and Adolescent Child Health Research Program, Kenya Medical Research Institute, Nairobi, Kenya

**Keywords:** Cervical squamous intraepithelial lesions, prevalence, human immunodeficiency virus, Kenya

## Abstract

**Introduction:**

cervical intraepithelial neoplasia the precursor of cervical cancer occurs with increased frequency in women infected with human immunodeficiency virus (HIV). This study aimed at determining the prevalence and correlates of abnormal cervical cytology among HIV-infected women and compare to the uninfected women.

**Methods:**

a cross-sectional study conducted among HIV-infected and uninfected women enrolled in a HIV study in Central Kenya. All women had baseline Pap smear examination assessed using Bethesda system. Bivariate and multivariate logistic regression methods were employed to assess the correlates of cervical squamous epithelial lesions (CSIL).

**Results:**

a total 480 women had an acceptable baseline smear, 373 (78%) were HIV-infected. Median age was 30.2 years [IQR 25.4-35.5]. Overall prevalence of CSIL was 37% (176/480) with the prevalence of low grade squamous intraepithelial lesion (LSIL), atypical squamous cells undetermined significance (ASCUS), high grade squamous intraepithelial lesions (HSIL) and atypical glandular cells (AGC) were 17%, 14%, 4% and 2% respectively. HIV-infected women had a higher prevalence of CSIL at 42% as compared to HIV-uninfected women at 19%. HIV infection was the predictor associated with development of CSIL at multivariate analysis and specifically, HIV-infected women were 3 times (AOR 3.1, 95% CI: 1.8 - 5.4, p<0.005) more likely to have CSIL than HIV-uninfected women. The age 35 - 44 years was protective to developing CSIL (AOR 0.45, 95% CI: 0.24 - 0.87, p=0.018).

**Conclusion:**

cervical squamous epithelial lesions is a major problem among Kenyan women. HIV infection confers a higher risk to development of CSIL. Cervical cancer screening should be an established practice in HIV programs.

## Introduction

Cervical cancer was the fourth most frequently diagnosed cancer and the 4^th^ leading cause of cancer death in women with an estimated 570,000 cases and 311,000 deaths worldwide in 2018 [1]. However, in many sub-Saharan African countries, it is the most commonly diagnosed and the leading cause of cancer deaths among women. The 2018 estimates indicate 5,250 women were diagnosed with cervical cancer in Kenya; it is the 2^nd^ leading cause of female cancer and the 1^st^ most common female cancer in women aged 15 and 44 years [2]. Despite the magnitude of the problem in Kenya and the fact that it's easily preventable, the cervical cancer screening coverage for all women 15 to 49 years of age in Kenya is estimated at 14% [3].

The areas where cervical cancer rates are highest often have high prevalence of HIV and the presence of HIV increases the risk of cervical precancerous and cancerous changes; furthermore, there is general unavailability of effective cervical cancer screening programs in these lower resource settings. In 2018, the HIV prevalence in Kenya among adults was estimated at 4.9%, with the prevalence being twice as high among women at 6.6% compared to men at 3.1% [4]. HIV-infected women have higher prevalence of human papillomavirus (HPV), higher incidence of HPV [5,6], higher HPV viral load [7], longer persistence of HPV [8], higher likelihood of multiple HPV subtypes [9] and greater prevalence of oncogenic subtypes [10], than HIV-uninfected women.

The prevalence and incidence of abnormal Pap smears are increased among HIV-infected women as compared to uninfected women, with up to 10-fold higher rates [11]. The role of HIV in the etiology of invasive cervical cancer especially in Africa is not conclusive. As is the case with HIV-negative women, oncogenic HPV types play a central role in the relationship between HIV and cervical cancer. Our involvement in undertaking research on HIV prevention among HIV sero-discordant couples in Central Kenya, offered us the opportunity to investigate the prevalence and correlates of cervical squamous intraepithelial lesions and therefore, give recommendations on to cervical screening programs in Kenya.

## Methods

**Study setting:** the study site was the partners in prevention house in Thika, Kiambu County (Central Kenya) which was one of the study sites for the ethically approved Partners Pre-exposure Prophylaxis (PrEP) Study (Partners PrEP Study) titled *“Parallel comparison of tenofovir and emtricitabine/tenofovir pre-exposure prophylaxis to prevent HIV-1 acquisition within HIV-1 discordant couples”* [12].

**Study design:** this was a cross-sectional study conducted at baseline and was part of an ongoing cohort study in the partners PrEP study with a minimum follow up of 3 years from December 2008. Data collection was done at the time of screening using standardized questionnaires from study participants.

**Study population:** a total of 495 HIV sero-discordant couples were consented and enrolled into the study. One partner was HIV-1 infected and the other was uninfected aged between 18 and 65 years. The cohort was established through recruitment efforts in the community from partnering voluntary counseling and testing centers (VCT), outreach workers, public promotion of couple´s HIV testing by well-known opinion leaders and community organizations such as churches.

**Screening and follow up:** HIV-uninfected women were followed up on a monthly basis given that they were taking PrEP and required close follow-up, while the HIV-infected women every 3 months. At enrollment, HIV-infected women were not receiving antiretroviral therapy (ART) and did not meet Kenyan guidelines for initiation of ART. At the baseline visit, data on socio-demographics, behavioral information, sexually transmitted infections (STI) testing through polymerase chain reaction (PCR) and Papanicolaou testing (Pap smear) test were done for all 495 women regardless of HIV status. The HIV-uninfected women being on study medication would be tested for HIV on a monthly basis while 6-monthly CD4 counts testing was done for the HIV-infected women. Those who became eligible for initiation of ART according to national guidelines were actively counseled to initiate treatment and referred to local clinics. All women had Pap smear testing and STI screening for (syphilis, gonorrhea, chlamydia and trichomonas) annually. Women with genital symptoms were examined and treated accordingly. Both symptomatic and laboratory confirmed (PCR-based) genitourinary infections would be treated as per STI treatment guidelines. Pap smear specimens were taken for cervical cytology. They would be read and classified by a pathologist using the 2001 Bethesda classification. Smear readers were not blinded to previous smear results but every smear was verified by a second reader.

**Management of cervical SIL:** women with abnormal cytology results were immediately traced for follow-up or referred for colposcopy. Women diagnosed with low-grade squamous intraepithelial lesion/cervical intraepithelial neoplasia (LSIL/CIN I) and atypical squamous cells undetermined significance (ASCUS) were carefully followed-up and a repeat cytological smear was obtained six months following initial diagnosis. Women diagnosed with high-grade squamous intra-epithelial lesion (HSIL/CIN II/III) and atypical glandular cells of undetermined significance (AGUS) at the time of cervical cytology or at the time of colposcopy, would have a cervical biopsy taken during the colposcopy to make a definitive diagnosis. Those confirmed with (CIN II/III) by histology would receive standard care with loop electrosurgical excision procedure (LEEP). Any diagnosis of invasive cervical cancer was referred for standard care. The study covered all the costs for all screening and standard care treatment provided within the clinic. Having a cervical biopsy done, heightened the risk of acquiring or transmitting HIV, therefore women were strongly advised to refrain from sex for 10 days. They were also instructed to use condoms to reduce HIV risk acquisition/transmission if abstaining was not possible. Those who underwent LEEP were strongly counseled to refrain from sex for 6 weeks and instructed to use condoms to reduce HIV risk acquisition/transmission if abstaining was not possible.

**Data collection:** data on demographic and reproductive characteristics, HIV status and follow up, STI status and treatment and Pap smear results and follow up were collected in case report forms and electronic databases within the partners PrEP study.

**Statistical analysis:** de-identified data from case report forms was entered in excel worksheets and exported into STATA version 12 (StataCorp LLC, College Station, TX, 77845 USA) for data analysis. Baseline characteristics were presented using frequencies and percentages stratified by HIV status. Bivariate and multivariable logistic regression analysis was performed to determine factors associated with cervical intraepithelial lesions. Crude and adjusted odds ratio (AOR) at 95% CI were presented with a p-value threshold of P<0.005 set for statistical significance.

**Ethical approval:** the partners PrEP study protocol was approved by the Kenyatta National Hospital - University of Nairobi Ethics and Research Committee.

## Results

**Socio-demographic characteristics of study participants:** a total of 495 women enrolled in the study, 15 women (10 HIV-infected and 5 uninfected) were excluded because the smears were not adequate for evaluation, leaving 480 for further analysis. Of these, 373 (78%) were HIV-infected and 107 (22%) were HIV-uninfected. At enrollment, none of the HIV-infected women was on anti-retroviral therapy or qualified for ART based on the national guidelines then. The median age of the participants was 30.2 years [IQR 25.4-35.5]. A third (34%) had no formal education. Two thirds of the women (67%) had 2 or more children. Majority of the women (96%) had an average monthly income of ≤ Ksh. 4,999 (< USD 50) and 14% had a diagnosis of an STI or genital inflammatory condition at enrollment. Two hundred and twenty-three (46%) women were not using any form of contraception (Table 1).

**Table 1:** socio-demographic and reproductive characteristics of women participating in a HIV prevention study in Central Kenya stratified by HIV status

Characteristics	HIV Negative n (%)	HIV Positive n (%)	Total n (%)
**Age, years**			
≤ 24	11(12)	78(88)	89(18)
25-34	55(21)	202(79)	257(54)
35-44	27(27)	73(73)	100(21)
≥45	14(41)	20(59)	34(7)
**Marital status**			
No	2(9)	21(91)	23(5)
Yes	105(23)	352(77)	457(95)
**Education level**			
None	36(22)	127(78)	163(34)
Primary	29(18)	130(82)	159(33)
Secondary	34(26)	96(74)	130(27)
Tertiary/ university	8(29)	20(71)	28(6)
**Number of children**			
No child	4(10)	38(90)	42(9)
1 child	17(15)	99(85)	116(24)
2 or more children	86(27)	236(73)	322(67)
**Income level**			
≤Ksh. 4,999	102(22)	359(78)	461(96)
Ksh. 5,000 - 9,999	5(29)	12(71)	17(3.5)
Ksh. 10,000 - 14,999	0(0)	1(100)	1(<1)
≥Ksh. 15,000	0(0)	1(100)	(<1)
**Diagnosis of STI/other genital inflammatory conditions**			
Yes	10(15)	56(85)	66(14)
No	97(23)	317(77)	414(86)
**Contraception status use at baseline**			
None	52(23)	171(77)	223(46)
Pregnant	0(0)	53(100)	53(11)
On any form of contraception	55(27)	149(73)	204 (43)

HIV: human immunodeficiency virus; STI: sexually transmitted infection

**Prevalence of cervical squamous intraepithelial lesions:** at baseline, 304 (63%) women had a normal smear as compared to 176 (37%) who had abnormal smears. A total of 87 (81%) of HIV-uninfected women had a normal smear as compared to 20 (19%) with abnormal Pap smears. Among HIV-infected women, 217 (58%) had a normal smear compared to 156 (42%) with abnormal smear. Overall prevalence of cervical squamous intraepithelial lesions in this cohort was (37%), with a higher prevalence among HIV-infected women (42%), of which the prevalence of ASCUS -16% (n = 60), LSIL -18% (n = 68), HSIL - 5% (n = 18), AGUS - 3% (n = 10) as compared to the prevalence of 19% among HIV un-infected women 19%, with a prevalence of 6% (n = 6), 11% (n = 12), 1% (n = 1) and 1% (n = 1) for ASCUS, LSIL, HSIL and AGUS respectively p < 0.005. There was no diagnosis of cervical squamous cell carcinoma (Table 2).

**Table 2:** prevalence of squamous intraepithelial lesions of the cervix in HIV-infected and uninfected women in Central Kenya at their baseline cervical smear based on Bethesda classification (n=480)

	Total	HIV-infected	HIV-uninfected	p value
	n (%)	n (%)	n (%)	
Normal smears	304 (63)	217 (58)	87 (81)	<0.005
Cervical squamous intraepithelial lesions (CSIL)	176 (37)	156 (42)	20 (19)	
ASCUS	66 (14)	60 (16)	6 (6)	
LSIL	80 (17)	68 (18)	12 (11)	
HSIL	19 (4)	18 (5)	1 (1)	
AGUS	11 (2)	10 (3)	1 (1)	
Cancer	0	0	0	

AGUS: atypical glandular cells of atypical significance; ASCUS: atypical squamous cells of uncertain significance; CSIL: cervical squamous intraepithelial lesion; HIV: human immunodeficiency virus; HSIL: high grade squamous intraepithelial lesion; LSIL: low grade squamous intraepithelial lesion

**Correlates for cervical squamous intraepithelial lesions:** bivariate and multivariate logistic regression analyses were employed to determine association with cervical squamous intraepithelial lesions. With both bivariate and multivariate analysis, the risk of developing any cervical squamous lesion decreased within the age group of 35-44 years (AOR 0.45, 95% CI: 0.24-0.87, p = 0.018), while HIV-infected women were at high risk - 3 times higher of developing cervical SIL when compared to their HIV-uninfected counterparts (AOR 3.1, 95% CI: 1.8 - 5.4, p < 0.005) (Table 3).

**Table 3:** bivariate and multivariate analysis of risk factors for cervical squamous intraepithelial lesions among women screened for cervical cancer in a HIV prevention study in Central Kenya

Variable	CSIL		COR (95% CI)	P value	AOR (95% CI)	P value
	Positive n (%)	Negative n (%)				
**Age (years)**						
≤ 24	39 (44)	50 (56)	Ref		Ref	
25-34	98 (38)	159 (62)	0.79 (0.48-1.29)	0.345	0.74 (0.43-1.23)	0.248
35-44	28 (28)	72 (72)	0.49 (0.27-0.91)	0.024	0.45 (0.24-0.87)	0.018
≥45	11 (32)	23 (68)	0.61 (0.27-1.41)	0.249	0.61 (0.24-1.51)	0.283
**Marital status**						
No	9 (39)	14 (61)	Ref		Ref	
Yes	167 (37)	290 (63)	0.89 (0.3-2.1)	0.802	0.93 (0.37-2.33)	0.883
**No. of children**						
0	14 (33)	28 (67)	Ref		Ref	
1	41 (35)	75 (65)	1.09 (0.5-2.3)	0.815	1.15 (0.52-2.52)	0.733
≥ 2	121 (38)	201 (62)	1.2 (0 .6-2.3)	0.593	1.60 (0.76-3.39)	0.219
**Level of education**						
Tertiary	6 (21)	22 (79)	Ref		Ref	
None	60 (37)	103 (63)	2.13 (0.8-5.5)	0.12	1.82 (0.67-4.97)	0.243
Primary	58 (36)	101 (64)	2.1 (0.8-5.4)	0.128	1.73 (0.64-4.73)	0.283
Secondary	52 (40)	78 (60)	2.4 (0.9-6.4)	0.070	2.30 (0.83-6.35)	0.106
**Vaginal douching**						
No	65 (33)	134 (67)	Ref		Ref	
Yes	111 (40)	170 (60)	1.3 (0.9-1.9)	0.126	1.33 (0.89-1.98)	0.155
**STI**						
No	28 (42)	38 (58)	Ref		Ref	
Yes	148 (36)	266 (64)	1.3 (0.7-2.2)	0.297	1.27 (0.74-2.21)	0.388
**HIV status**						
Negative	156 (42)	217 (58)	Ref		Ref	
Positive	20 (19)	87 (81)	3.1 (1.8- 5.3)	0.000	3.13 (1.82-5.40)	<0.005

CSIL: cervical squamous intraepithelial lesion; HIV: human immunodeficiency virus; STI: sexually transmitted infection; CI: confidence interval

## Discussion

In this sample of Kenyan women, shows high prevalence of cervical squamous intra-epithelial lesions 37% with higher rates (42%) among HIV-infected women and (19%) in HIV-uninfected women. This depicts that squamous intra-epithelial lesions of the cervix are a significant health problem and more common in HIV-infected women than their uninfected counterparts. Low-grade lesions were more prevalent (LSIL - 17%, ASCUS - 14%) than high-grade ones (HSIL - 4%, AGUS - 2%). An important finding was that none of the cervical smears showed cytological features of carcinoma. Similarly, high rates of prevalent cervical squamous intraepithelial lesions in HIV-infected women have been reported in Kenya at 47% and 14% among HIV-uninfected women consisting of ASCUS - 20%, LSIL - 21%, HSIL - 5.8% [13] and in Soweto, South Africa among HIV-infected women at 38% [14] and the US at 38% among HIV-infected women and 16% among HIV-uninfected women [15]. Lower rates of cervical intra-epithelial lesions among HIV-infected women have been reported in similar settings of central Kenya - 26.7% [16], Tanzania 26.8% [17] and Ethiopia 17.8% [18]. This is likely due to differences in study populations such as smaller sample size and age. Higher prevalence rates observed in our study and others in South Africa and Kenya may be due to inclusion of a high number of HIV-infected women.

Our study demonstrated that the risk for cervical intra-epithelial lesions increases with HIV infection by more than 3 times (AOR 3.1, 95% CI: 1.8 - 5.4, p < 0.005). A study in Kenya reported that cervical lesions were associated with HPV infection and high-risk HPV infection among HIV-infected women as compared to uninfected women [13]. Other studies reveal that HIV induced immunosuppression increases the persistence of HPV infection [8], greater prevalence of oncogenic subtypes [10] and reactivation of HPV infection [19] than in HIV-uninfected women. The progression of untreated HPV-induced cervical dysplasia can lead to invasive cervical cancer which is an AIDS defining illness [20].

In our study, women in the age group of 35 - 44 years were less likely to develop cervical intra-epithelial lesions (AOR 0.45, 95% CI: 0.24 - 0.87, p = 0.018). A study from Rwanda reported similar findings of participants aged (41-50 years) being at lower risk of invasive and precancerous lesions [21]. This is in contrast to other studies that report women of 30 years and older were at great risk of developing cervical epithelial cell abnormality [18]. After accounting for classical risk factors in multivariate models such as marital status, parity, level of education, history of vaginal douching and diagnosis of STI, none were significant predictors of cervical intra-epithelial lesions.

This study had several limitations. The study was a convenience sample of women participating in an HIV prevention trial and therefore the prevalence of cervical intra-epithelial lesions in HIV-uninfected women may not be generalizable to the whole of Kenya. There was selection bias of HIV-infected women given that most women were recruited through antenatal clinics and couples voluntary counselling testing centres. There were limited variables available for analysis limiting the number of risk factors - no. of sexual partners, sexual debut age and confounders to study and moreover, the study did not assess the aetiology of CSIL. However, the study does allow for future analysis of progression of CSIL given the duration of follow-up.

## Conclusion

In this study, the prevalence of cervical intra-epithelial lesions among HIV-infected women was 42% which is among the highest rates reported in Africa. Women infected with HIV are at great risk of developing cervical intra-epithelial lesions than HIV-uninfected women. Therefore, regular screening of HIV-infected women should be prioritized. The study may not define an appropriate interval for screening but supports Kenya's recommendation that women should undergo early cervical cancer screening, with re-screening at least once a year and the introduction of HPV vaccine in the immunization program in prevention of cervical cancer whose precursor are cervical intra-epithelial lesions.

### What is known about this topic

- Cervical intraepithelial lesions are the precursor of cervical cancer;
- HIV increases the risk of developing cervical intra-epithelial lesions.

### What this study adds

- Affirms the need for regular screening for HIV-infected women and therefore the importance of integrating of cervical cancer screening in HIV programs;
- Central Kenya has among the highest rates of cervical intra-epithelial lesions among HIV-infected women in Africa (42%);
- Women in the age group 35-44 years have reduced risk of developing cervical intra-epithelial lesions.
